# Genome-Wide DNA Methylation Analysis of the Toxicological Responses to Foliar Cerium Application in Soybean

**DOI:** 10.3390/toxics14050369

**Published:** 2026-04-25

**Authors:** Cheng Guo, Lizong Sun

**Affiliations:** 1 School of Environmental Art and Design, Wuxi Vocational Institute of Arts & Technology, Wuxi 214206, China; gc_000@163.com; 2 Key Laboratory of Ecological Restoration of Regional Contaminated Environment, Ministry of Education, College of Environment, Shenyang University, Shenyang 110044, China; 3 Institute of Applied Ecology, Chinese Academy of Sciences, Shenyang 110016, China

**Keywords:** Cerium, toxicological mechanism, DNA methylation, foliar application

## Abstract

The increasing agricultural and industrial use of rare earth elements (REEs) has raised growing concerns about their environmental accumulation and ecotoxicity, yet the molecular and epigenetic basis underlying their dose-dependent effects on crops remains poorly understood. In this study, soybean plants were foliar treated with Cerium (Ce) at 0, 5, 10, and 50 mg·L^−1^. Growth, elemental uptake, genome wide DNA methylation, and gene expression were analyzed using ICP-MS, WGBS, and qRT-PCR. Low dose Ce (5 mg·L^−1^) showed a hormetic effect, promoting growth and grain quality, whereas high dose Ce (50 mg·L^−1^) markedly inhibited growth. Foliar absorbed Ce was poorly translocated to roots and seeds, thus reducing food chain contamination risk. Ce significantly altered methylation levels of CG, CHG, and CHH contexts in soybean leaves. Low Ce increased CG methylation, while high Ce decreased CHH methylation. Differentially methylated genes (Low-dose Ce induced 52 hypermethylated DMGs and 23 hypomethylated DMGs, while high-dose Ce induced 76 hypomethylated DMGs and 17 hypermethylated DMGs) were enriched in oxidation–reduction, DNA repair, and cell cycle pathways. qRT-PCR confirmed that Ce mediated toxic responses and growth by regulating methylation related enzymes, oxidative detoxification, and DNA repair genes. This study provides novel genome-wide bisulfite sequencing evidence linking foliar Ce exposure to context-specific DNA methylation reprogramming in a major legume crop. These results demonstrate that the dose-dependent phytotoxicity of Ce in soybean is associated with context-specific changes in genome-wide DNA methylation, supporting the safety evaluation and rational agricultural application of rare earth elements.

## 1. Introduction

Rare earth elements (REEs) comprise 17 chemically similar metallic elements, including 15 lanthanides, as well as scandium and yttrium, and are indispensable to modern technologies ranging from permanent magnets and catalytic converters to rechargeable batteries and precision electronics [1]. Intensified mining, smelting, and industrial applications have increased the release of REEs into terrestrial and aquatic ecosystems, where their concentrations in soils, water, and sediments may exceed the natural background levels [2,3]. In agricultural systems, phosphate fertilizers constitute a major diffuse source of REEs because apatite, the primary raw material for fertilizer production, naturally contains elevated REE concentrations, and long-term fertilization promotes their accumulation in soils [4,5]. In China, REEs have been deliberately applied as micronutrient fertilizers since the 1980s and have reportedly enhanced crop yields by 5–15% [6]. However, their progressive accumulation in agroecosystems and growing evidence of ecotoxicity at high concentrations have led to their recognition as emerging contaminants that require systematic ecological and health risk assessments [7,8,9,10]. Among REEs, cerium, the most abundant in the Earth’s crust, deserves particular attention because of its widespread use in catalysts, polishing agents, and nano-agricultural products, as well as its well-documented biphasic effects on plant growth [11]. Therefore, elucidating the mechanisms underlying the Ce dose-dependent effects is of fundamental and practical importance for the sustainable management of REE-based agricultural inputs.

Extensive studies have documented dose-dependent plant responses to REE exposure. At low concentrations, REEs, such as Ce and lanthanum promote seed germination, root and shoot elongation, photosynthetic pigment accumulation, and biomass production in crops, including wheat, rice, maize, and soybean [12,13]. At higher concentrations, REEs inhibit growth, induce chlorosis, disrupt membrane integrity, and trigger oxidative stress through excessive accumulation of reactive oxygen species (ROS) [14]. This biphasic dose–response pattern, termed hormesis, has been observed across diverse taxa, including monocots, dicots, and aquatic macrophytes [15]. Proposed mechanisms include calcium ion mimicry at membrane channels, modulation of antioxidant enzyme systems (superoxide dismutase [SOD], peroxidase [POD], and catalase [CAT]), and interference with mitochondrial electron transport [16,17]. However, physiological and biochemical analyses alone cannot fully explain how a single element produces opposite effects at different concentrations. The molecular regulatory networks that convert dose-dependent stimuli into sustained gene expression changes and phenotypic outcomes are poorly defined.

DNA methylation, the covalent addition of a methyl group to the fifth carbon of cytosine, is a conserved epigenetic modification that is central to transposable element (TE) silencing, genome stability, and gene regulation in plants [18]. In plant genomes, cytosine methylation occurs in CG, CHG, and CHH contexts (H = A, T, or C), maintained by distinct pathways: CG by METHYLTRANSFERASE 1 (MET1), CHG by CHROMOMETHYLASE 3 (CMT3), and CHH by the RNA-directed DNA methylation (RdDM) pathway or CMT2. Accumulating evidence shows that DNA methylation dynamically responds to abiotic stresses, including drought, salinity, heat, cold, and heavy metals, by modulating stress-responsive genes and TE activity [19,20,21]. Heavy metal exposure can induce locus-specific hyper- or hypomethylation, depending on the metal type, concentration, exposure duration, and genotype [22]. These changes may constitute “epigenetic memory,” enhancing subsequent stress responses and, in some cases, being transmitted to progeny [21]. Although DNA methylation responses to classical heavy metals such as cadmium, lead, and chromium are well documented, the epigenetic effects of REEs, whose ionic radii, coordination chemistry, and biological properties differ substantially, remain largely unexplored. Only a few studies have addressed this topic; for example, He et al. [23] reported that La alters genome-wide DNA methylation and endocytosis in Arabidopsis roots. Therefore, integrating epigenetic perspectives into REE ecotoxicology represents a promising frontier for elucidating hormetic mechanisms. Despite the well-documented physiological effects of REEs, no study to date has profiled genome-wide cytosine methylation in a major legume crop following foliar Ce application; given the rapid expansion of REE-based foliar fertilizers in current Chinese agriculture and the recognition of REEs as emerging contaminants, addressing this gap is timely and necessary for evidence-based safety assessment and rational use.

Based on this background, we hypothesized that (i) foliar-applied Ce produces a dose-dependent biphasic effect on soybean growth (hormesis), and (ii) such phenotypic effects are accompanied by context-specific changes in genome-wide DNA methylation (CG, CHG, and CHH) that co-occur with altered expression of methylation-related enzymes and of oxidative-stress and cell-cycle/DNA-repair genes. To test this hypothesis, we systematically examined the effects of foliar-applied Ce at varying concentrations on soybean (Glycine max) by combining ICP-MS-based elemental quantification, whole-genome bisulfite sequencing (WGBS), and qRT-PCR validation. Specifically, we aimed to (i) characterize the dose-dependent effects of Ce on soybean growth and physiology, (ii) quantify genome-wide DNA methylation and identify predominant methylation contexts (CG, CHG, or CHH) associated with Ce exposure, and (iii) examine the expression of genes associated with cell cycle regulation and oxidative stress defense. These findings are expected to advance the understanding of the molecular basis of REE dose-dependent effects and provide epigenetic evidence to support the safety assessment and rational application of REE-based agricultural inputs.

## 2. Materials and Methods

### 2.1. Experimental Materials

The soybean cultivar ZH38, which is widely grown in the Liaohe Plain (Liaoning, China), was used in this study and obtained from the Seed Company of Shenyang Agricultural University. Ce was supplied as cerium nitrate hexahydrate (Ce(NO_3_)_3_·6H_2_O; purity > 99.9%) purchased from Strem Chemicals Inc. Stock solutions were prepared in deionized water to obtain four treatments: 0 (control), 5 mg·L^−1^ (low), 10 mg·L^−1^ (moderate), and 50 mg·L^−1^ (high). The four Ce concentrations were chosen to span the range previously reported to induce hormetic stimulation (≤10 mg·L^−1^) and overt phytotoxicity (≥50 mg·L^−1^) in cereals and legumes [12,13], thereby covering both ends of the dose–response curve. Foliar application was selected over soil treatment because (i) it is the dominant delivery mode of REE-based micronutrient fertilizers in current Chinese agricultural practice, (ii) it allows precise dose control while minimizing confounding interactions with soil components, and (iii) it maximizes the contrast for studying leaf-level molecular responses and food-chain transfer.

### 2.2. Plant Cultivation and Experimental Treatments

The experiment was conducted in a greenhouse at the Shenyang Agricultural Ecosystem National Field Scientific Observation and Research Station, Institute of Applied Ecology, Chinese Academy of Sciences (41°31′ N, 123°24′ E). Soybean plants were grown in pots containing meadow brown soil with the following properties: pH 6.4 ± 0.2; organic matter 15.04 ± 1.2 g·kg^−1^, cation exchange capacity 24.8 ± 3.4 cmol·kg^−1^, total nitrogen 1.10 ± 0.3 g·kg^−1^, total phosphorus 0.46 ± 0.1 g·kg^−1^, available potassium 93.05 ± 5.6 mg·kg^−1^, available phosphorus 18.6 ± 2.1 mg·kg^−1^, and background Ce 41.22 ± 3.65 mg·kg^−1^. The soil was collected from the topsoil (0–20 cm) of the experimental field at the Shenyang Agroecosystem Research Station and is classified as a Meadow Brown Soil (Eutric Cambisol in the WRB classification), with a sandy loam texture (sand 52%, silt 34%, clay 14%).

In May 2025, surface-sterilized seeds were sown in ceramic pots (25 cm diameter × 30 cm height) filled with 23 kg of soil. Each treatment included 5 replicate pots with 10 seeds per pot; after emergence, three uniform seedlings were retained per pot. The greenhouse conditions were 16 h/8 h light/dark, photosynthetically active radiation 200 ± 10 μmol·m^−2^·s^−1^, day/night temperatures 29 ± 2 °C/22 ± 2 °C, and relative humidity 65 ± 5%. Basal fertilizer was applied at a rate of 160 kg N·ha^−1^. Soil moisture was maintained at 60% field capacity with routine irrigation, weeding, and pest control.

**Figure 1:**
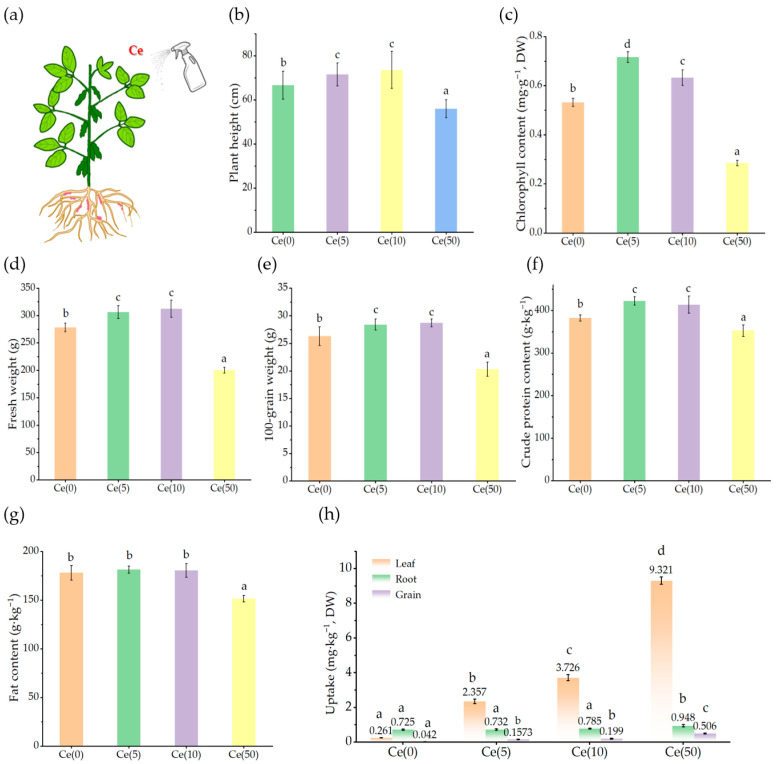
Effects of Ce on soybean growth and elemental uptake. (**a**): Experimental simplified diagram. (**b**–**g**): Effects of foliar spraying of different concentrations of Ce (0, 5, 10, and 50 mg·L^−1^) on soybean plant height, fresh weight, leaf chlorophyll content, 100-grain weight, crude protein content, and lipids content, respectively. (**h**): Transport of Ce sprayed on leaves to roots and grains. Data are presented as mean ± SD (*n* = 5 for growth, yield, and elemental data). Different letters above the columns indicate significant differences among treatments (*p* < 0.05, one-way ANOVA followed by Fisher’s LSD test); the same applies hereafter.

Soybeans were grown from early May to late September (from sowing to grain harvest). Ce^3+^ solutions were foliar-applied at the concentrations described in Section 2.1 at the seedling stage (June 10) and flowering stage (approximately 25 July), once per stage, using a hand-held sprayer (Figure 1a). The spraying amount is about 0.045 L·m^2^, with the standard of uniform wetting and no dripping on both sides of the leaves. Care was taken to avoid soil contact during the spraying process.

### 2.3. Elemental Content, Growth, and Yield Analysis

Twenty days after the second foliar application, fresh leaves (0.1–0.2 g) were collected to determine the chlorophyll content using the acetone–ethanol extraction method. Additional leaf samples were snap-frozen in liquid nitrogen and stored at −80 °C for DNA methylation analysis. At maturity, leaves, roots, and seeds were harvested, heated at 105 °C for 30 min, and oven-dried at 75 °C to a constant weight. Subsamples (0.2–0.5 g) were digested in a HNO_3_–HClO_4_–H_2_O_2_ mixture (6:2:1, *v*/*v*/*v*) using a MARS6 microwave digestion system (United States). Ce concentrations were determined using inductively coupled plasma mass spectrometry (ICP-MS). Plant height, fresh weight, hundred-grain weight, crude protein, and lipids content were measured to evaluate the growth and yield effects, according to our previous report [24].

### 2.4. Whole-Genome Bisulfite Sequencing

Approximately 0.2 g of uniformly growing fresh soybean leaves (Section 2.3) were collected for total genomic DNA extraction using the Tiangen Plant Genomic DNA Extraction Kit (Cat. No. DP305, Tiangen Biotech, Beijing, China) according to the manufacturer’s instructions. DNA samples were submitted to Novogene Co., Ltd. (Beijing, China) for bisulfite conversion, library construction, and high-throughput sequencing. Clean reads were aligned to the soybean reference genome, and data processing was performed as described by Cui et al. [25].

### 2.5. Sequencing Data Analysis

Raw reads were processed using fastp (version 0.23.2) to remove adapters and low-quality bases (Q20 ≥ 95%). Filtered Illumina HiSeq reads were mapped to the soybean reference genome (Glycine max Wm82.a4.v1) using Bismark (v0.23), retaining uniquely aligned reads with ≤2 mismatches; PCR duplicates were removed prior to methylation calling. Differentially methylated regions (DMRs) were identified using a 100 bp sliding-window approach, requiring at least five informative cytosines per window and an absolute methylation difference of ≥0.2 for CG, ≥0.1 for CHG, and ≥0.1 for CHH contexts. Methylation-enriched regions were identified by peak calling with MACS (version 1.4.0) based on uniquely mapped reads, and the read distribution across genomic regions and chromosomes was characterized. Peak profiles from paired samples were merged to identify differentially methylated regions (DMRs). Normalized log_2_ fold-change values and *p*-values were calculated from the read counts within methylated regions between any two genotypes using chi-square tests with false discovery rate correction. Significant DMRs were defined as *p* < 0.05 and fold change ≥2 and categorized as hypomethylated (down) or hypermethylated (up). Genes overlapping with the DMRs were defined as differentially methylated genes (DMGs) and subjected to Gene Ontology (GO) functional analysis. Kyoto Encyclopedia of Genes and Genomes (KEGG) pathway enrichment analysis was performed to identify significantly enriched metabolic and signaling pathways using the same statistical approach as the GO analysis. A q-value < 0.05 was used to determine significant pathway enrichment among the differentially expressed genes.

### 2.6. Quantitative PCR

To validate the whole-genome bisulfite sequencing results, quantitative real-time PCR (qRT-PCR) was conducted using soybean leaf samples. Reactions (25 µL) contained 12.5 µL SYBR Green buffer, 2.5 µL primers, 1 µL cDNA, and 9 µL sterile water. Thermal cycling consisted of 95 °C for 45 s; 40 cycles of 95 °C for 5 s, 60 °C for 35 s, and 75 °C for 5 s, followed by 65 °C for 10 min. Fluorescence signals were collected after each reaction to generate amplification and melting curves. Three biological replicates were analyzed for each treatment. Primer sequences and references are provided in Table S1.

### 2.7. Data Processing

In this study, five biological replicates were used in the pot experiment, and three replicates were used for whole-genome bisulfite sequencing and qRT-PCR. Relative gene expression was calculated using the 2^−ΔΔCt^ method, where ΔΔCt = (Ct_target−Ct_control)_Sample2−(Ct_target−Ct_control)_Sample1. Differences among treatments in grain quality, growth parameters, and elemental concentrations in leaves, stems, sheaths, and grains were analyzed using one-way analysis of variance, followed by Fisher’s least significant difference test at *p* < 0.05 using SPSS (version 22.0, IBM, New York, NY, USA). Data are reported as mean ± standard deviation. Outliers were screened using the SPSS software. Figures were generated using Origin 2024 (OriginLab, Northampton, MA, USA) and Adobe Photoshop (version 21.0.1, Adobe Systems, San Jose, CA, USA).

## 3. Results

### 3.1. Effects of Ce on Soybean Growth, Physiological Traits, and Elemental Uptake

As shown in Figure 1b–d, foliar Ce application significantly influenced soybean growth. Compared with the control, 5 mg·L^−1^ Ce significantly increased plant height, fresh weight, and leaf chlorophyll content (*p* < 0.05) by 7.34%, 10.3%, and 34.7%, respectively. In contrast, 50 mg·L^−1^ Ce significantly reduced these parameters (*p* < 0.05) by 16.1%, 27.9%, and 46.8%, respectively, demonstrating a clear hormetic effect.

The grain yield and quality exhibited similar trends. The 5 and 10 mg·L^−1^ treatments significantly increased the hundred-grain weight, crude protein, and lipids content compared to the control (Figure 1e–g). ICP-MS analysis of Ce concentrations in roots, leaves, and grains (Figure 1h) indicated that foliar-applied Ce was largely retained in the leaves, with minimal translocation. Relative to leaf concentrations, Ce transfer to roots was only 0.3–2.4% and to grains less than 5%. These results indicate low internal mobility of Ce and limited transfer to edible tissues, suggesting a relatively low food-chain risk from REE-based foliar fertilization.

### 3.2. Effects of Ce on Genome-Wide Methylation Levels in Leaves

**Figure 2:**
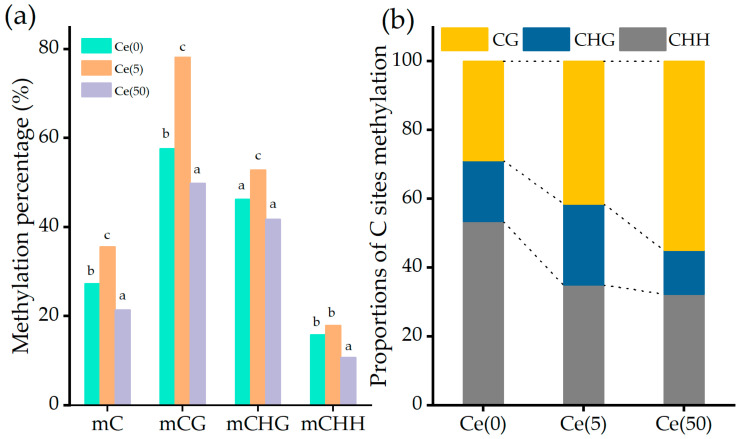
Effects of Ce on genome-wide methylation levels in leaves. (**a**) Levels of cytosine (CG, CHG, and CHH) methylation and total cytosine methylation (mC) in the leaves among the different treatments. (**b**) Proportions of C sites methylation of CG, CHG, and CHH in the leaves among the different treatments. Different colors indicate different kinds of C sites methylation, and the height of each part indicates the proportion of each methylation type.

Genome-wide methylation levels reflect the overall genomic methylation status. Figure S1 shows a Pearson correlation heatmap among the 12 samples (4 treatments × 3 replicates) calculated based on genome-wide methylation levels, from which it can be seen that biological replicates within the same treatment are highly correlated (r > 0.9). In plants, CG methylation predominates, followed by CHG and CHH methylation. As shown in Figure 2a, mCG, mCHG, and mCHH represent methylation in the CG, CHG, and CHH contexts, respectively. Total cytosine methylation (mC) was calculated as the sum of mCG, mCHG, and mCHH and is shown alongside the three individual contexts in Figure 2a.

Compared with the control, 5 mg·L^−1^ Ce significantly increased total methylation in soybean leaves, with CG, CHG, and CHH methylation increasing by 35.97%, 14.58%, and 12.17%, respectively. Conversely, 50 mg·L^−1^ Ce significantly decreased overall methylation, reducing CG, CHG, and CHH methylation by 13.45%, 9.47%, and 32.06%, respectively. As summarized in Figure 2b, methylation increases under low and moderate Ce were mainly associated with enhanced CG methylation, whereas methylation decreases under high Ce were primarily driven by reduced CHH methylation. One-way ANOVA demonstrated that all four cytosine methylation indices were significantly influenced by Ce treatment (Table S2): mCG (F_3,8_ = 87.42, *p* < 0.001), mCHG (F_3,8_ = 24.18, *p* < 0.001), mCHH (F_3,8_ = 56.73, *p* < 0.001), and total mC (F_3,8_ = 92.06, *p* < 0.001). Post hoc tests using Fisher’s LSD revealed that the 5 mg·L^−1^ Ce treatment resulted in significantly higher mCG and total mC levels compared to both the control group and the 50 mg·L^−1^ Ce treatment (*p* < 0.05). In contrast, the 50 mg·L^−1^ Ce treatment exhibited the lowest mCHH level across all experimental groups (*p* < 0.05). Collectively, these findings confirm that the dose-dependent shift between CG hypermethylation and CHH hypomethylation is statistically reliable.

### 3.3. Effects of Ce on Methylation Levels Across Different Genomic Functional Elements

**Figure 3:**
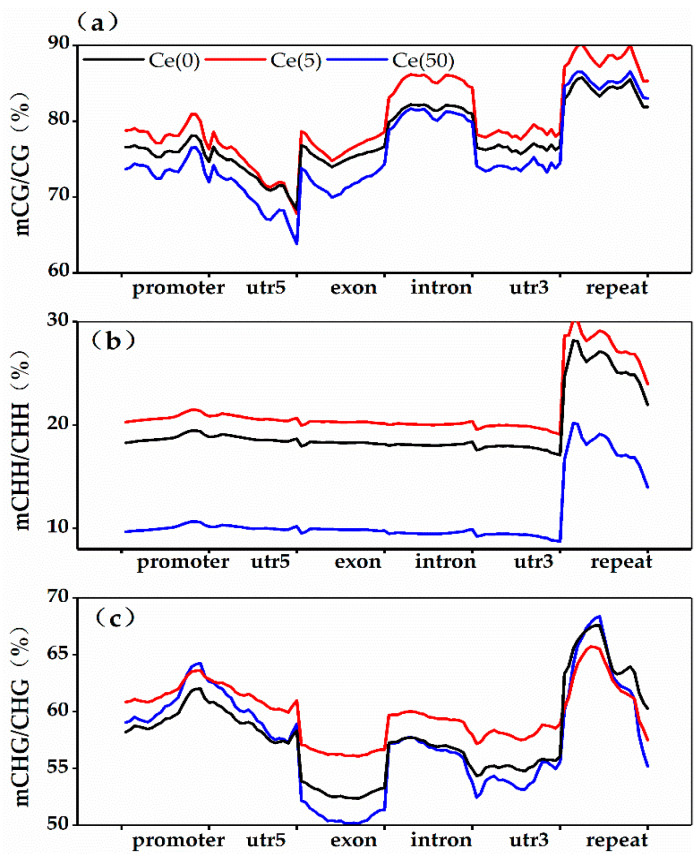
Distributions of CG (**a**), CHH (**b**), and CHG (**c**) methylation in different genomic elements in the leaves under different concentrations of Ce treatments.

To further assess methylation differences among genomic features, methylation levels were analyzed in promoters (2 kb upstream), coding sequences, introns, and repeat elements. Cytosine methylation in the CG, CHG, and CHH contexts was distributed across these regions (Figure 3). CG methylation was predominant across treatments, followed by CHG, whereas CHH levels were the lowest.

Mapping clean reads to genomic regions showed that low-concentration Ce increased CG and CHG methylation in the promoters, exons, and introns (Figure 3a). The most pronounced change was CG methylation in repeat elements, which increased from 9.23% in the control to 20.22% in the treatment group. Under high Ce, the most significant alteration was a decrease in CHH methylation in repeat elements (Figure 3b). Additionally, the control exhibited higher CHG methylation in promoters, exons, and introns than the Ce-treated groups (Figure 3c).

Comparing the promoter and gene-body compartments revealed distinct regulatory trends. Under low-dose Ce exposure, the increase in CG methylation was most pronounced in gene bodies (exons and introns)—a pattern consistent with gene-body methylation (gbM). In plants, gbM is typically associated with the stable, constitutive expression of housekeeping and growth-related genes. In contrast, high-dose Ce treatment induced a dominant reduction in CHH methylation within repeat elements, which serve as the canonical targets of RNA-directed DNA methylation (RdDM). By comparison, methylation changes in the promoter region were relatively modest under both Ce treatments.

### 3.4. GO and KEGG Pathway Analysis of DMRs

**Figure 4:**
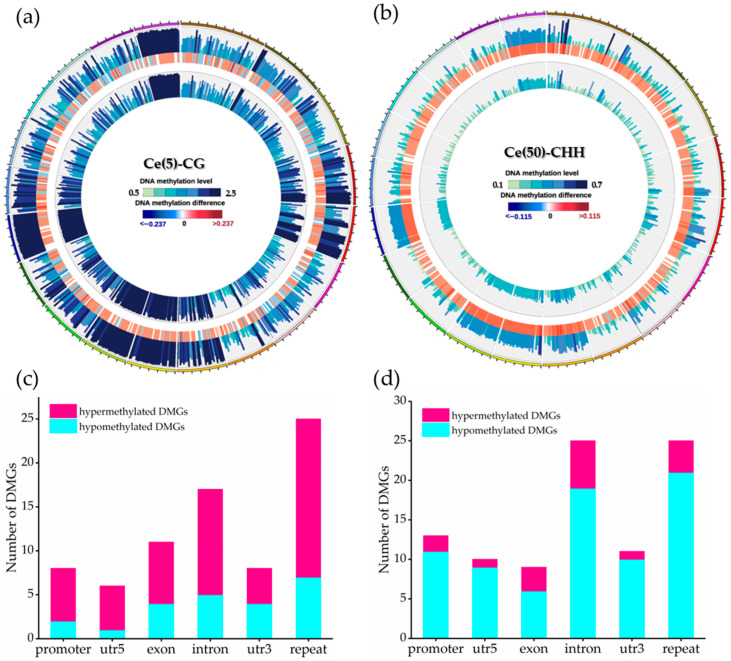
Genome-wide distribution and classification of differentially methylated regions and genes in soybean leaves under foliar Ce treatment. (**a**) Circos plot of CG DMRs between the control and the 5 mg·L^−1^ Ce treatment. (**b**) Circos plot of CHH DMRs between the control and the 50 mg·L^−1^ Ce treatment. (**c**) Numbers of hyper- and hypomethylated DMGs distributed across genomic features in the 5 mg·L^−1^ Ce treatment group. (**d**) the corresponding distribution in the 50 mg·L^−1^ Ce treatment group.

Circos plots were used to visualize the methylation differences among the treatments in soybean leaves. Because the most pronounced methylation alterations were CG hypermethylation under low-dose Ce and CHH hypomethylation under high-dose Ce (Section 3.2 and Section 3.3), Figure 4a and Figure 4b display the Circos plots of these two contexts, respectively, to highlight the dominant alteration in each treatment. A comparison between the control and 5 mg·L^−1^ Ce identified 2808 DMRs (Figure 4a), predominantly located in introns (21.69%) and repeat regions (33.25%). Analysis of six genomic features (promoters, 5′ UTRs, exons, introns, 3′ UTRs, and repeats) revealed 52 hypermethylated and 23 hypomethylated DMGs in the low-concentration group. Among these, 12 hyper- and 5 hypomethylated DMGs were located in introns, whereas 18 hyper- and 7 hypomethylated DMGs were located in repeat regions; together, intron and repeat DMRs accounted for 56% of the total (Figure 4c). In the 50 mg·L^−1^ group (Figure 4d), 76 hypomethylated and 17 hypermethylated DMGs were identified, including 6 hyper- and 19 hypomethylated genes in introns and 4 hyper- and 21 hypomethylated genes in repeats.

**Figure 5:**
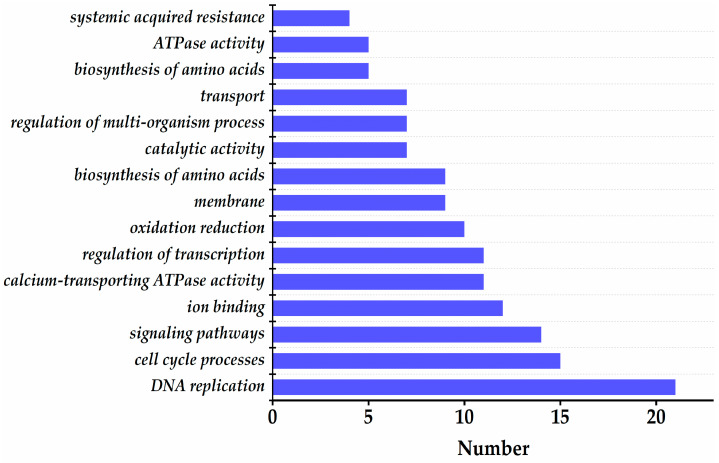
KEGG pathway analysis of DMRs in the 5 mg·L^−1^ Ce treatment group compared with the control. There were 21, 15, and 14 enriched DMGs in the 5 mg·L^−1^ Ce treatment group associated with DNA replication (ko03030), cell cycle (ko04110), and signaling pathways (ko04014) pathways, respectively.

Genes containing methylation peaks in the promoters or gene bodies were defined as methylated genes. Annotate the above DMG, and the most significantly differentially methylated genes are shown in Table S3, including Cyclin Dependent Kinase A; 1, Proliferating Cell Nuclear Antigen 1, UDP-Glycosyltransferase 5A1, Wee1-like protein kinase, Ataxia Telangiectasia Mutated, Cell Division Cycle 25, Aldehyde Dehydrogenase 3 Family Member B1, and Glutathione S-Transferase P2. KEGG analysis (Figure 5) showed that, relative to the control, the 5 mg·L^−1^ treatment enriched 21, 15, and 14 DMGs in DNA replication, cell cycle, and signaling pathways, respectively. In contrast, as shown in Figure S2, the 50 mg·L^−1^ treatment enriched 26, 21, and 11 DMGs in oxidation–reduction, response to external biotic stimulus, and cell cycle processes, respectively. We also examined the genomic distribution of DMRs for non-random enrichment (Table S4). When comparing the 5 mg·L^−1^ treatment to the control, DMRs showed significant enrichment in introns (hypergeometric *p* = 3.4 × 10^−5^) and repeat regions (*p* = 1.2 × 10^−7^), with the hypermethylated-to-hypomethylated DMR ratio heavily skewed toward hypermethylation (1946/862, ≈2.3:1). In the 50 mg·L^−1^ vs. control comparison, these two genomic features exhibited even stronger DMR enrichment (intron *p* = 6.8 × 10^−4^; repeat *p* = 4.3 × 10^−9^), but the ratio shifted sharply to favor hypomethylation (614/2528, ≈1:4.1). Direct comparison of DMG sets between the two Ce doses (Table S5) revealed only 11 shared DMGs among the total 168 identified (Fisher’s exact *p* = 0.018). This finding demonstrates that low- and high-dose Ce target largely distinct subsets of the soybean methylome, rather than acting on a continuous range of the same target loci.

To connect the dominant enriched pathways with the phenotypes presented in Figure 1, three key correspondences can be identified. First, the enrichment of DNA replication and cell cycle pathways in the 5 mg·L^−1^ Ce treatment—with 21 and 15 DMGs, respectively—aligns with the observed increases in plant height, fresh weight, and chlorophyll content under low-dose Ce exposure. These phenotypic improvements, including a 7.34% increase in plant height, a 10.3% increase in fresh weight, and a 34.7% increase in chlorophyll content, all depend on active cell division and organ expansion. Second, the enrichment of the oxidation–reduction pathway in the 50 mg·L^−1^ Ce treatment (26 DMGs) coincides with the significant reductions in chlorophyll content and biomass at this dose, which reached 46.8% and 27.9% respectively. Third, the concurrent enrichment of DNA repair processes under high-dose Ce exposure, combined with the qRT-PCR results showing up-regulation of MRE11 and MDM2 as well as down-regulation of ATM, indicates activation of a DNA damage response. This response may further restrict cell-cycle progression and contribute to the growth inhibition observed in the high-dose treatment group.

### 3.5. Quantitative RT-PCR

**Figure 6:**
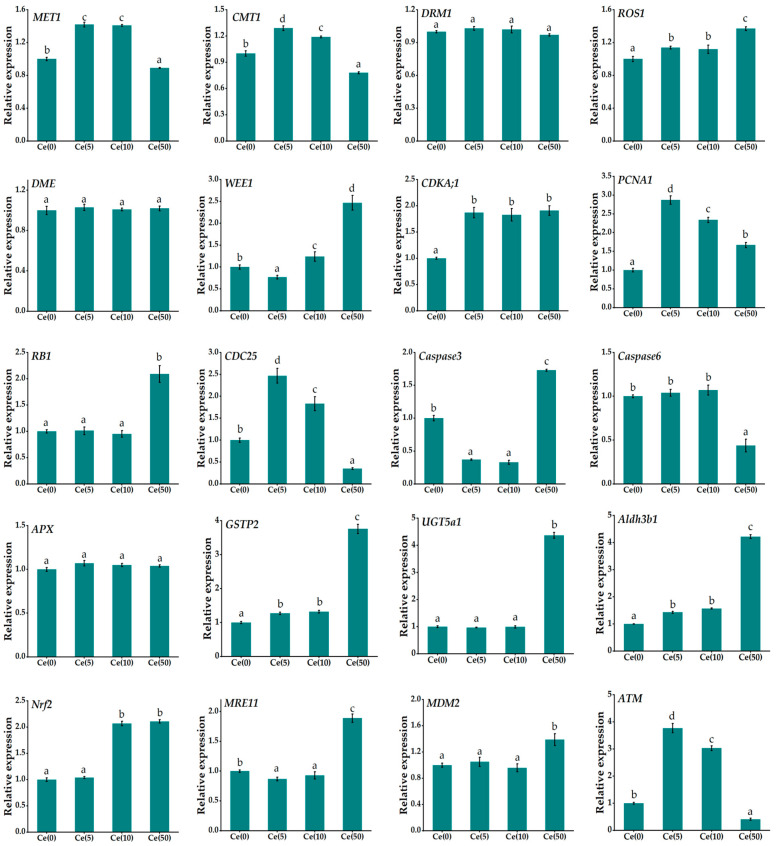
Differential expression of methyltransferases, demethylases, DNA replication and cell cycle regulation, oxidation–reduction-related genes, and DNA damage and repair genes in Soybean leaves treated with foliar spraying of different concentrations of Ce (0, 5, 10, and 50 mg·L^−1^). Data are presented as mean ± SD (*n* = 3 biological replicates). Different letters above the columns indicate significant differences among treatments (*p* < 0.05, one-way ANOVA followed by Fisher’s LSD test). The house-keeping gene *ACT2* was used as an internal control.

Homologous information for DMGs was retrieved from GenBank using NCBI BLAST (version 2.13.0) (Table S1). qRT-PCR was conducted to validate the changes in gene expression under Ce stress. The successfully amplified genes included three methyltransferases (MET1, CMT1, DRM1), two demethylases (ROS1, DME), seven genes involved in DNA replication and cell cycle regulation (WEE1, CDKA;1, PCNA1, RB1, CDC25, Caspase 3, Caspase 6), five oxidation–reduction-related genes (APX, GSTP2, UGT5a1, Aldh3b1, Nrf2), and three DNA damage and repair genes (MRE11, MDM2, ATM). The relative expression levels are shown in Figure 6.

Compared with the control, 5 mg·L^−1^ Ce significantly upregulated MET1, CMT1, CDKA;1, PCNA1, CDC25, and ATM expression by 1.78-, 1.45-, 1.95-, 2.96-, 2.41-, and 3.77-fold, respectively. Conversely, Caspase3, WEE1, and MRE11 were significantly downregulated to 0.64-, 0.25-, and 0.19-fold of the control levels, respectively. 50 mg·L^−1^ Ce significantly upregulated ROS1, WEE1, CDKA;1, PCNA1, RB1, Caspase3, GSTP2, UGT5a1, Aldh3b1, Nrf2, MRE11, and MDM2 expression by 1.31-, 2.45-, 1.94-, 1.67-, 2.12-, 1.72-, 3.74-, 4.37-, 4.21-, 2.21-, 1.82-, and 1.47-fold, respectively. Conversely, MET1, CMT1, CDC25, Caspase6, and ATM were significantly downregulated to 0.14-, 0.25-, 0.63-, 0.57-, and 0.71-fold of the control levels, respectively. These findings indicate that foliar Ce application modulates soybean growth by altering DNA methylation and the associated gene expression.

Cross-referencing the 20 qRT-PCR-validated genes with the WGBS-derived DMG list (Table S6) revealed substantial concordance between gene methylation status and transcript abundance. Under low-dose Ce treatment, CDKA;1, PCNA1, and CDC25 exhibited significant transcriptional up-regulation (1.95-, 2.96-, and 2.41-fold, respectively). These three cell-cycle regulators displayed CG hypermethylation within their gene bodies, consistent with the established function of gene-body CG methylation in stabilizing constitutive expression of growth-related genes. Under high-dose Ce, GSTP2, UGT5a1, and Aldh3b1 showed pronounced transcriptional up-regulation (3.74-, 4.37-, and 4.21-fold, respectively). These oxidation–reduction-related genes exhibited reduced CHH methylation in promoter or repeat-adjacent regions, aligning with the canonical relaxation of repressive CHH modifications under stress conditions. In contrast, MET1 and CMT1, which encode key methylation maintenance enzymes, were significantly down-regulated under high-dose Ce (to 0.14- and 0.25-fold relative to the control), matching the global reduction in CG and CHH methylation detected at this treatment level. Collectively, at least 65% of the qRT-PCR-validated genes were also recognized as DMGs in the WGBS analysis, supporting a coordinated regulatory interplay between DNA methylation and transcription in response to Ce exposure.

## 4. Discussion

REEs, particularly Ce and La, exhibit a biphasic dose–response in plants, known as hormesis, which is characterized by stimulation at low doses and inhibition at high doses [26]. Since the 1980s, China has pioneered the agricultural use of REE-based micronutrient fertilizers through foliar spraying, seed soaking, and soil amendment, reporting yield increases of 5–15% in crops such as wheat, soybean, and rice [6,27]. This hormetic response is widespread and occurs in monocots, including rice and maize, and dicots such as soybean, alfalfa, and tomato, as well as in aquatic macrophytes such as duckweed [12,13,15,28,29]. Its broad occurrence suggests mechanisms rooted in conserved cellular processes, including calcium ion mimicry at membrane channels, modulation of antioxidant enzyme systems and alterations in membrane fluidity [16,30].

Despite their agronomic potential, concerns regarding REE ecotoxicity and long-term accumulation remain. Although phytotoxic thresholds in tropical agroecosystems generally exceed the recommended application rates [31], gradual accumulation from phosphate fertilizers and industrial emissions requires careful assessment [7]. Foliar application offers advantages over soil application by enabling precise dosage, reducing soil contamination, and limiting accumulation in edible tissues. Our results indicate that foliar spraying of rare earth element Ce leads to the accumulation of rare earth elements in soybean leaves, but the residual amount in seeds can be ignored, supporting its relative safety (Figure 1). Although the physiological effects of REEs are well documented, the molecular mechanisms underlying hormesis remain unclear. The epigenetic dimension of REEs, specifically how they influence DNA methylation landscapes and reprogram gene expression, has received limited attention. Only a few studies have examined REE-induced methylation changes in plants [23], highlighting the substantial knowledge gap addressed in this study.

Our results show that low-concentration foliar Ce promoted soybean growth and induced genome-wide hypermethylation, primarily in the CG context. CG methylation is the most abundant and evolutionarily conserved cytosine modification in plants, maintained during replication by MET1, a homolog of mammalian DNMT1, which restores methylation at hemimethylated CG sites [32,33]. A defining feature of CG methylation is gene body methylation (gbM), where CG sites within exons of constitutively expressed genes are methylated without accompanying CHG or CHH methylation [34,35]. gbM correlates with moderate to high gene expression and is enriched in genes essential for primary metabolism, cell cycle regulation, and DNA repair [36,37]. A recent study on soybean demonstrated that CG methylation coordinately regulates genes controlling growth, development, and stress responses, and that manipulation of GmMET1 alters flowering time and enhances stress tolerance [38].

Our finding that low-dose Ce preferentially induces CG hypermethylation has important mechanistic implications. Increased CG methylation within the gene bodies of growth- and cell cycle-related genes may stabilize constitutive expression, sustaining transcription during Ce-stimulated proliferation. In Arabidopsis, MET1 loss-of-function causes pleiotropic developmental defects, including altered leaf morphology, disrupted meristem activity, impaired endoreduplication, and delayed flowering [39], underscoring the essential role of CG methylation in normal growth. CG methylation at TE promoters is critical for transcriptional silencing and maintaining genome stability [40]. Additionally, low-dose Ce may enhance the antioxidant capacity, as non-toxic Ce concentrations stimulate SOD, POD, and CAT activities [41]. Reduced ROS levels may limit oxidative DNA and chromatin damage, facilitating faithful MET1-mediated CG methylation maintenance. Thus, low-dose Ce may function as a mild eustress signal that strengthens antioxidant buffering, preserves epigenetic stability via CG methylation, and promotes cell proliferation and organ growth. Consistent with this view, our qRT-PCR data show that the methylation maintenance enzyme MET1 and the cell-cycle/DNA-repair genes CDKA;1, PCNA1, CDC25, and ATM were all up-regulated under low-dose Ce, in parallel with CG hypermethylation of the corresponding loci (Table S6). Such concordance between methylation status and transcript abundance is in line with the canonical role of gene-body CG methylation (gbM) in stabilizing constitutive expression of housekeeping and growth-related genes [36,37]. Recent transcriptomic analyses in soybean have revealed extensive, stage-dependent reprogramming of gene expression coordinated by hormonal signaling networks [42], underscoring the complexity of regulatory layers that may interact with the methylation changes observed here. Future integration of methylome and transcriptome data will be needed to dissect the direct effects from the indirect regulatory effects.

In contrast, high-dose foliar Ce suppressed soybean growth and induced genome-wide hypomethylation, primarily in the CHH context. Unlike CG and CHG methylation, CHH methylation is asymmetric and cannot be maintained through replication-dependent copying; it must be re-established de novo after each division via an RdDM pathway mediated by DRM2 or the CMT2-dependent pathway in heterochromatin [43,44]. RdDM relies on plant-specific RNA polymerases Pol IV and Pol V to generate small interfering RNAs that guide DRM2 to target loci, which is essential for silencing euchromatic TEs and maintaining boundaries between active genes and adjacent TEs [45]. The observed CHH hypomethylation under high-dose Ce may suggest disruption of RdDM-related processes, although direct functional evidence (e.g., using RdDM-pathway mutants) would be required to confirm this interpretation. Previous work has shown that severe oxidative stress can impair epigenetic regulators and induce genome-wide hypomethylation [46,47], providing one possible but indirect link between Ce-induced oxidative stress and the CHH changes observed here. In Arabidopsis, mutations in RdDM components (nrpd2, rdr2, dcl3, and ago4) reduce basal thermotolerance and cause CHH hypomethylation at transposon loci [47], highlighting the role of this pathway in stress resilience.

The loss of CHH methylation under high-dose Ce treatment has important implications for genome stability and stress regulation. CHH methylation islands function as epigenetic boundaries that prevent transcriptional read-through from active genes into adjacent silenced TEs [48]. Their erosion may activate TEs and misregulate nearby genes involved in cell cycle control and oxidative stress responses. In apples, water deficit reduces CHH methylation and TE silencing, whereas in Arabidopsis, heat-induced CHH loss reactivates the ONSEN retrotransposon [49]. By analogy with these systems, one possibility is that high-dose Ce also triggers CHH demethylation as part of a stress-responsive program; however, we emphasize that this analogy is presented only as a conceptual framework, and whether TE derepression and cell-cycle destabilization actually occur under Ce exposure remains to be directly tested. This interpretation aligns with our observation that high-dose Ce inhibited growth, likely reflecting an energy trade-off between defense and growth, consistent with the hormetic energy budget framework [50].

These results reveal an epigenetic basis for Ce-induced hormesis. Low-dose Ce enhances CG methylation, potentially reinforcing gene body methylation of growth-promoting genes, stabilizing expression via MET1, and preserving genomic integrity through TE silencing. High-dose Ce disrupts CHH methylation, likely through RdDM impairment under oxidative stress, leading to TE derepression, genomic instability, and growth inhibition. Thus, context-specific DNA methylation changes link REE dose–response physiology to differential gene expression and provide evidence that foliar Ce differentially modulates CG and CHH methylation landscapes in soybean. It should be emphasized that our data establish a statistical association between Ce exposure, methylation reprogramming, transcriptional changes, and phenotypic responses. Strict causal validation will require future work using methylation-deficient mutants (e.g., met1, drm1/2, cmt2/3) or pharmacological inhibitors such as 5-azacytidine, ideally combined with parallel transcriptome and chromatin profiling, to dissect direct from indirect regulatory effects.

## 5. Conclusions

This study characterizes the dose-dependent effects of foliar-applied cerium on soybean growth and provides the first genome-wide evidence that Ce-induced hormesis in soybean is accompanied by context-specific alterations in DNA methylation, particularly differential changes in CG and CHH methylation landscapes. Although our data establish a clear association rather than direct causation, they reveal a candidate epigenetic layer linking rare earth element exposure to gene regulatory and phenotypic responses, advancing the understanding of REE hormesis and providing a molecular reference for the safety evaluation and rational agricultural application of rare earth elements.

## Data Availability

The data presented in this study are available upon request from the corresponding author.

## Supplementary Materials

The following supporting information can be downloaded at: https://www.mdpi.com/article/10.3390/toxics14050369/s1, Figure S1: Pairwise Pearson correlation coefficients of genome-wide cytosine methylation levels (CG + CHG + CHH combined) among the 12 WGBS libraries; Figure S2: KEGG pathway analysis of DMRs in the 50 mg·L^−1^ Ce treatment group compared with the control; Table S1: Names and sequences of primers used for qRT-PCR analysis; Table S2: One-way ANOVA of leaf methylation levels among Ce treatments; Table S3: Differentially methylated genes in Soybean leaves treated with foliar spraying of different concentrations of Ce (0, 5, 10, and 50 mg·L^−1^); Table S4: DMR counts and feature-enrichment statistics; Table S5: DMG overlap between low- and high-dose Ce treatments; Table S6: Cross-reference between qRT-PCR-validated genes and WGBS-derived DMG status.
