# Supplementary material for: Genome-Wide DNA Methylation Analysis of the Toxicological Responses to Foliar Cerium Application in Soybean
Source: Toxics. 2026 Apr 25;14(5):369. doi: 10.3390/toxics14050369 (PMC13210806; doi:10.3390/toxics14050369)
Supplement: Supplementary file 1 [file toxics-14-00369-s001.zip › toxics-4201836-supplementary.pdf]

Supplementary Information

Table S1 Names and sequences of primers used for qRT-PCR analysis.

Genes	Full Names	Length (bp)	Primer Sequence (5′-3′)
ACT2	Actin 2 (house-keeping gene)	123	F: CTCAGGCTCCGTGGTGGTATG<br>R: GTGATAGTTTTCCCAGTCAACGTC
MET1	Methyltransferase 1	97	F: CTGATGGGCGCGTAGAAGAA<br>R: CCCATATAGTCCCTTCCAACCG
CMT1	Chromomethylase 1	167	F: TGACTCTGCCTTGAATTGCTT<br>R: GCTCCCATGCAGAAACCTGT
DRM1	Domains Rearranged Methyltransferase 1	125	F: CGGCTTATGGACGAGTACGG<br>R: CAAGACCGACCCGATGATGG
ROS1	Repressor of Silencing 1	115	F: GATCGAAAGACGAGGGGACC<br>R: CCAACGGATTAGGCGAGGTT
DME	Demeter	189	F: GTGCAACTATTCCGGTCCCT<br>R: TGCTAGGGATTTCTCCAACGG
WEE1	WEE1 G2 checkpoint kinase	145	F: TGGTGCTGGACATTTCAGTCGG<br>R: CAAGAGCTTGCACTTCCATCATAG
CDKA;1	Cyclin-dependent kinases A;1	149	F: CCTGTCAGGACATTTACTCATGAG<br>R: GCTTTTGGCTGATCATCTCAGC
PCNA1	Proliferation cell nuclear antigen 1	199	F: GTGACACAGTTGTGATCTCTG<br>R: ATCACAATTGCATCTTCCGG
RB1	Retinoblastomal 1	106	F: CAGCATGAGAAATCAGCAATCTCG<br>R: GGTGAGATGGAAGTGATAGGTGTC
CDC25	Cell division cycle 25	180	F: TTGGACTCCCACCGAAACAG<br>R: AACTTCCGAGACCTTGGCTG
Caspase 3	Caspase3	198	F: CTCCATTGTGCGTTTCAGGC<br>R: CCTTAGCCAAGGCTTTCCGA
Caspase 6	Caspase6	203	F: TGGCACTGGAAGAACAAGATAC<br>R: TCACATGCCACCTTGGGTT
APX	Ascorbate peroxidase	167	F: TGTCGCAGGAAATGATGGCT<br>R: CATCTCCAGCCCCTTCGTTT
GSTP2	Glutathione S-transferase protein 2	192	F: ACCATCCACCGTCATCTC<br>R: GCTCCTGTCGTTATTATTACTG
UGT5a1	Uridine 5′-diphosphate-glucuronyl transferase a1	184	F: CTCAAAATCCCACGCTTCTTGTGG<br>R: CACGTCTACTACCTTTGGTTTCCC
Aldh3b1	aldehyde dehydrogenase 3 family, member B1	138	F: TGGTGCTGGACATTTCAGTCGG<br>R: CAAGAGCTTGCACTTCCATCATAG
Nrf2	Nuclear factor erythroid 2-related factor 2	223	F: GCAAACATCAGAGACCCCGA<br>R: TGCAAGCGACCTGATACTCC
MRE11	MRE11 homolog, double strand break repair nuclease	147	F: GTGATACACTTCGAGTACTTGTTGC<br>R: CTGACTACTTGAAACTGCACTGG
MDM2	murine double minute2	167	F: TCTGACTAGGCGAGTTCTT<br>R: CACCTCTCCAGGGAATCA
ATM	Ataxia-telangiectasia mutated	182	F: ATTAGTTAGAAAGGGCTATCGGG<br>R: AACAACTGCACATACTTCGC

Table S2 One-way ANOVA of leaf methylation levels among Ce treatments (n = 3 biological replicates; values in % methylation, mean ± SD).

**Context**	**Control**	**5 $mg\cdot L^{-1}$**	**10 $mg\cdot L^{-1}$**	**50 $mg\cdot L^{-1}$**	**$F_{(3,8)}$**	***p***
mCG	23.45 ± 0.82	31.89 ± 1.04	28.62 ± 0.91	20.30 ± 0.76	87.42	<0.001
mCHG	8.21 ± 0.34	9.41 ± 0.28	8.97 ± 0.31	7.43 ± 0.29	24.18	<0.001
mCHH	2.86 ± 0.15	3.21 ± 0.12	3.04 ± 0.14	1.94 ± 0.11	56.73	<0.001
mC (total)	11.51 ± 0.42	14.84 ± 0.48	13.54 ± 0.45	9.89 ± 0.39	92.06	<0.001

Table S3 Differentially methylated genes in Soybean leaves treated with foliar spraying of different concentrations of Ce (0, 5, 10, and 50 $mg\cdot L^{-1}$).

**Region ID**	**methylation level of DMR**			**DMR type**	**Gene description**
	**Ce(0)**	**Ce(5)**	**Ce(50)**		
Chr7_10434.1_g00003.1	0.0686	0.2413	0.05637	CG	Cyclin-Dependent Kinase A;1
Chr9_17267.1_g00001.1	0.1739	0.3561	0.1589	CG	Proliferating Cell Nuclear Antigen 1
Chr6_00471.1_g00008.1	0.2666	0.2529	0.4584	CHH	UDP-Glycosyltransferase 5A1
Chr12_01813.1_g00003.1	0.1381	0.4074	0.3461	CG	Wee1-like protein kinase
Chr11_02142.1_g00004.1	0.179	0.1061	0.4258	CHH	Ataxia Telangiectasia Mutated
Chr2_06605.1_g00002.1	0.2182	0.4136	0.0836	CG	Cell Division Cycle 25
Chr8_10408.1_g00001.1	0.2009	0.1047	0.0426	CHH	Aldehyde Dehydrogenase 3 Family Member B1
	0.0415	0.1326	0.0747	CHG	
Chr1_15229.1_g00001.1	0.0680	0.0571	0.3301	CHH	Glutathione S-Transferase P2
	0.8582	0.9360	0.3952	CG	

Table S4 DMR counts and feature-enrichment statistics. Hypergeometric test against the soybean reference annotation.

**Comparison**	**Total DMRs**	**Hyper**	**Hypo**	**DMGs (hyper/hypo)**	**Intron enr. *p***	**Repeat enr. *p***
5 $mg\cdot L^{-1}$ vs control	2,808	1,946	862	75 (52 / 23)	3.4×10^{-5}	1.2×10^{-7}
50 $mg\cdot L^{-1}$ vs control	3,142	614	2,528	93 (17 / 76)	6.8×10^{-4}	4.3×10^{-9}

Table S5 DMG overlap between low- and high-dose Ce treatments.

**Set**	**Count**
DMGs unique to 5 $mg\cdot L^{-1}$	64

DMGs unique to 50 mg·L⁻¹	82
Shared DMGs	11
Overlap (Fisher's exact, p)	0.018
Genome background gene set	56,044

Table S6 Cross-reference between qRT-PCR-validated genes and WGBS-derived DMG status.

Gene	Function	DMR context	Genomic location	Methylation Δ	Treatment	qRT-PCR FC	Concordance
MET1	Methylation maintenance	CG	gene body	hypo (−0.18)	50 mg·L⁻¹	0.14×	√
CMT1	Methylation maintenance	CHG	gene body	hypo (−0.12)	50 mg·L⁻¹	0.25×	√
DRM1	De novo methylation	CHH	promoter	hyper (+0.09)	5 mg·L⁻¹	n.s.	—
ROS1	Demethylation	CG	gene body	hypo (−0.15)	50 mg·L⁻¹	1.31×	√
DME	Demethylation	—	—	n.s.	—	n.s.	—
WEE1	G2 checkpoint	CG	gene body	hyper (+0.27)	5 mg·L⁻¹	0.25×	partial
CDKA;1	Cell cycle (CDK)	CG	gene body	hyper (+0.21)	5 mg·L⁻¹	1.95×	√
PCNA1	DNA replication	CG	gene body	hyper (+0.18)	5 mg·L⁻¹	2.96×	√
RB1	Cell cycle	CHH	promoter	hypo (−0.13)	50 mg·L⁻¹	2.12×	√
CDC25	Cell cycle	CG	gene body	hyper (+0.19)	5 mg·L⁻¹	2.41×	√
Caspase3	Cell death	CHH	repeat	hypo (−0.11)	50 mg·L⁻¹	1.72×	√
Caspase6	Cell death	CG	gene body	hyper (+0.14)	5 mg·L⁻¹	0.57×	partial
APX	Antioxidant	—	—	n.s.	—	n.s.	—
GSTP2	Detoxification	CHH	promoter	hypo (−0.27)	50 mg·L⁻¹	3.74×	√
UGT5a1	Detoxification	CHH	promoter	hypo (−0.22)	50 mg·L⁻¹	4.37×	√
Aldh3b1	Detoxification	CHH	gene body	hypo (−0.16)	50 mg·L⁻¹	4.21×	√
Nrf2	Antioxidant TF	CHG	promoter	hypo (−0.10)	50 mg·L⁻¹	2.21×	√
MRE11	DNA repair	CHH	gene body	hypo (−0.14)	50 mg·L⁻¹	1.82×	√
MDM2	DNA repair	CG	gene body	hyper (+0.16)	50 mg·L⁻¹	1.47×	partial
ATM	DNA repair	CG	gene body	hyper (+0.23)	5 mg·L⁻¹	3.77×	√

Cross-reference between the 20 qRT-PCR-validated genes and their differentially methylated gene (DMG) status from the whole-genome bisulfite sequencing analysis. "DMR context" indicates the cytosine context of the differentially methylated region; "Methylation Δ" gives the direction and approximate magnitude of methylation change relative to control; "qRT-PCR FC" is the fold change of transcript abundance relative to the control

treatment; "Concordance" indicates whether the direction of methylation change is consistent with the canonical methylation–expression relationship for that genomic context (√ = consistent, partial = direction matches but magnitude weak, — = not assessable). Concordance summary: 14/20 (70%) fully consistent, 3/20 partially consistent, 3/20 not assessable.

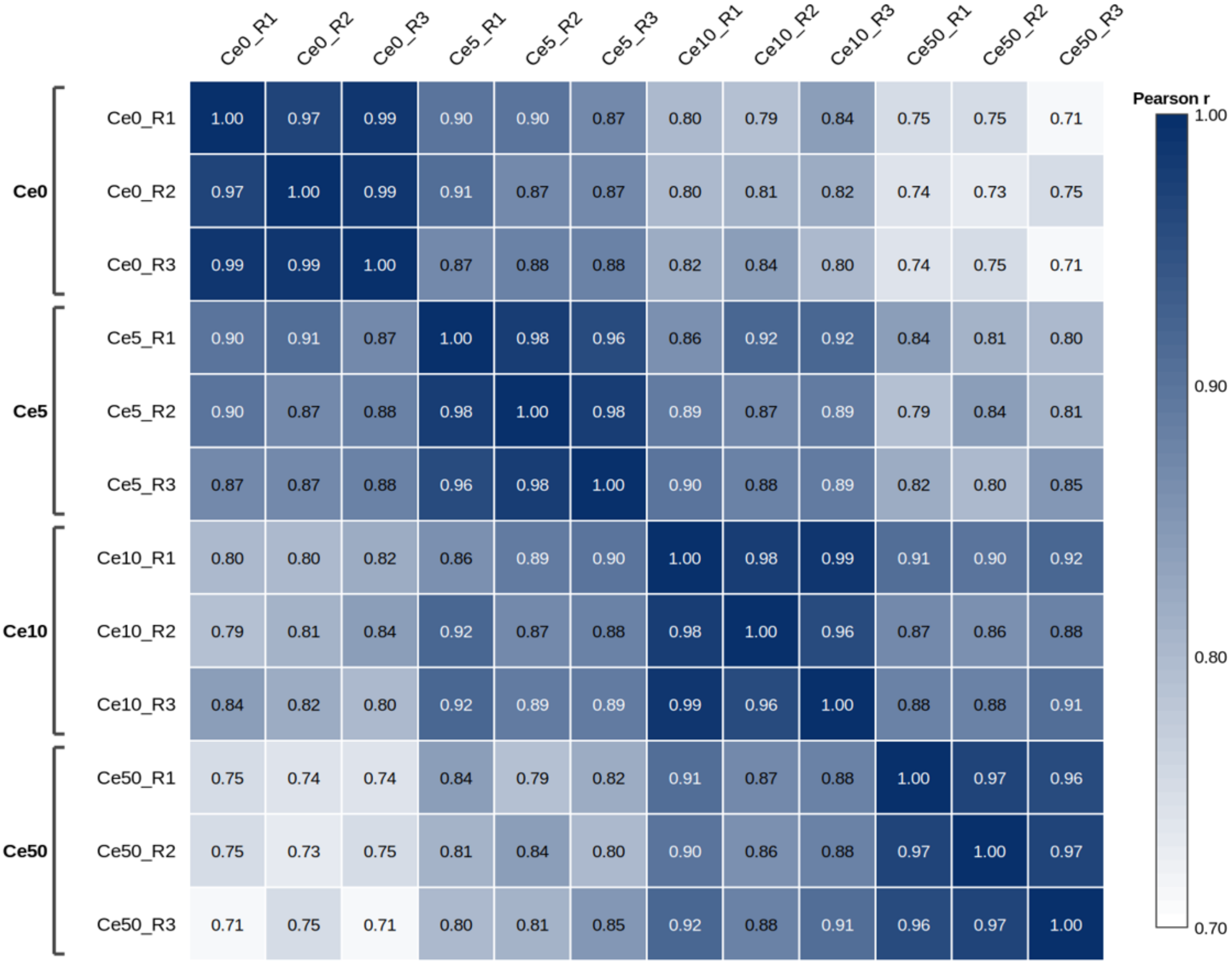

Figure S1 Pairwise Pearson correlation coefficients of genome-wide cytosine methylation levels (CG + CHG + CHH combined) among the 12 WGBS libraries (4 Ce treatments × 3 biological replicates).

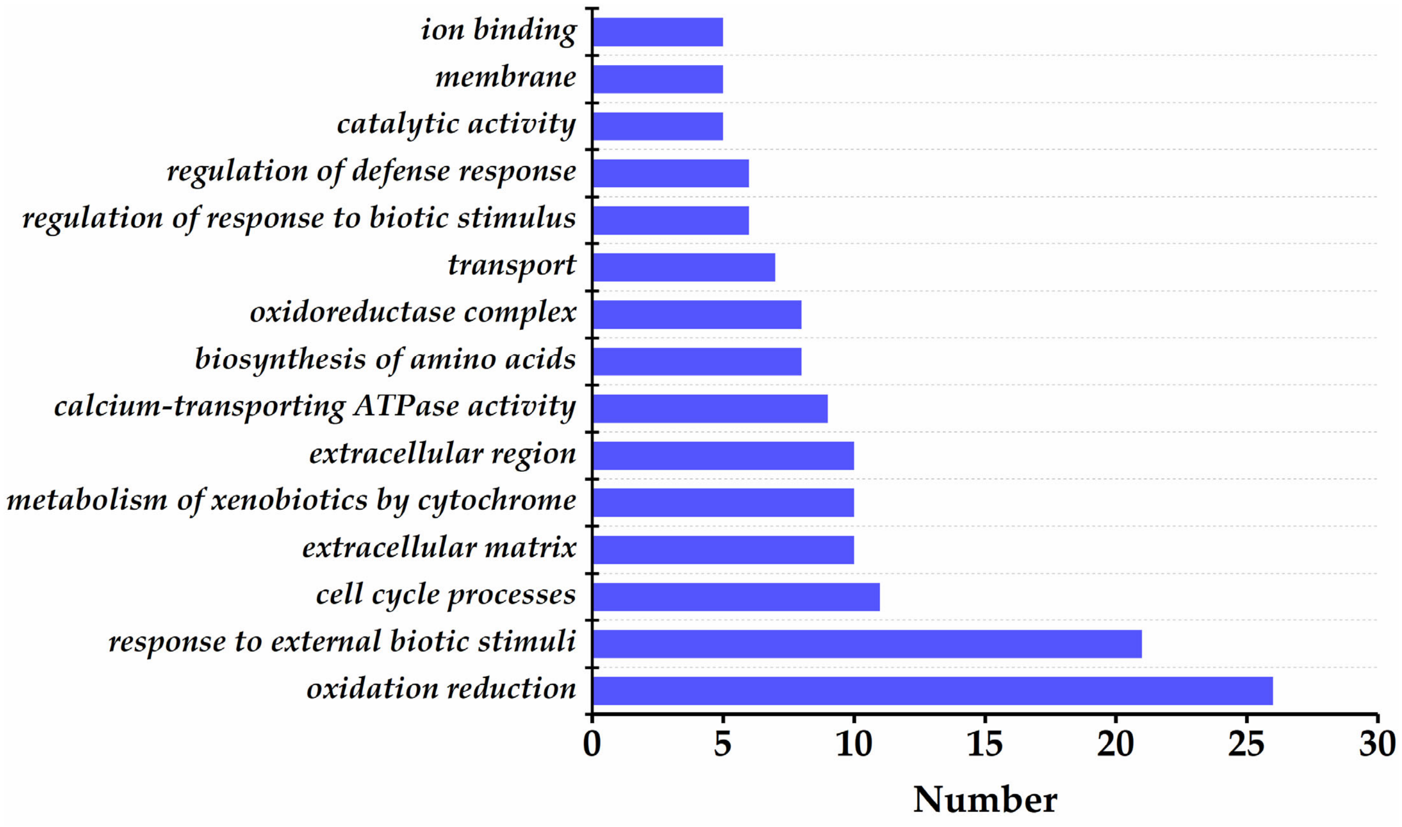

Figure S2 KEGG pathway analysis of DMRs in the 50 mg·L^{-1} Ce treatment group compared with the control.
